# Stage–specific incidence trends of renal cancers in the East of England, 1999–2016

**DOI:** 10.1016/j.canep.2020.101883

**Published:** 2021-04

**Authors:** Annie Herbert, Matthew E. Barclay, Minjoung M. Koo, Brian Rous, David C. Greenberg, Gary Abel, Georgios Lyratzopoulos

**Affiliations:** a Epidemiology of Cancer Healthcare and Outcomes (ECHO) Research Group, Department of Behavioural Science and Health, University College London, London, UK; b MRC Integrative Epidemiology Unit, Bristol Medical School, Department of Population Health Sciences, University of Bristol, Bristol, UK; c The Healthcare Improvement Studies (THIS) Institute, University of Cambridge, Cambridge, UK; d National Cancer Registration and Analysis Service (NCRAS), Public Health England, London, UK; e Medical School (Primary Care), University of Exeter, Exeter, UK

**Keywords:** Renal, Cancer, Stage, Diagnosis, Trends

## Abstract

**Objectives:**

To determine stage-specific time-trends in renal cancer incidence.

**Methods:**

We used population-based East Anglia data 1999−2016 (population ∼2 million) on 5,456 primary renal cancer diagnoses, estimating stage-specific annual incidence using Poisson regression, allowing for changing time-trends, and adjusting for sex, age, and socioeconomic deprivation.

**Results:**

Renal cancer incidence increased from 9.8–16.4 cases per 100,000 during 1999−2016. Incidence of Stage I, II, and III cases increased over time, most steeply for Stage I, with annual Incidence Rate Ratio [IRR] for Stage I of 1.09 (95 % CI 1.07–1.12) during 1999−2010; and 1.03 (1.00–1.05) during 2011−2016. In contrast, the annual incidence of Stage IV renal cancer decreased during most years, IRR of 0.99 (0.98–1.00) during 2003−2016.

**Conclusion:**

The findings are consistent with both earlier detection of symptomatic renal cancer and increasing identification of asymptomatic lesions. However, the decreasing incidence of late-stage disease suggests genuine shifts towards earlier diagnosis.

## Highlights

• Renal cancer incidence is increasing globally.
• The contribution of early or late stage disease in unclear.
• During 1999−2016 increasing incidence was chiefly driven by early stage disease.
• For most of the study period advanced stage renal cancer was deceasing.
• The findings are compatible with a range of interpretations.

## 1 Introduction

In different countries, in spite of decreasing mortality, the incidence of renal cancer has been increasing since the mid-1990s [[1], [2], [3]]. In the UK, renal cancer incidence has approximately doubled since 1995, with further increases projected over the next two decades [1]. Understanding the drivers of increasing incidence is important for informing relevant cancer prevention and control policies.

Rising trends in the incidence of renal cancer have been attributed to greater use of abdominal imaging over time, leading to increasing incidental detection of small asymptomatic tumours (i.e. < 4 cm), principally in US populations [[4], [5], [6], [7], [8]]. Rising incidence, however, may also reflect increasing frequency of symptomatic disease, due to greater exposure of the population to risk factors such as obesity in previous decades [9,10].

Stage-specific incidence trends can elucidate the drivers of rising overall incidence [7,8,11,12]. We therefore examined stage-specific incidence trends of renal cancer in an English geographically-defined population with uniquely (relative to other English populations) highly complete information on stage at diagnosis in historical cohorts of patients with renal cancer. We hypothesised that the increasing incidence of renal diagnoses overall could be partly attributed to increasing incidence of early-stage cases, though remaining equipoised as to the exact partitioning of incidence trends in early or late stage disease.

## 2 Methods

### 2.1 Data

We analysed anonymised data for individuals with incident renal cancer (International Classification of Diseases for Oncology -3rd edition code C64) aged 25 and older in the East of England Anglia region diagnosed during 1999−2016. Data from this sub-region include highly complete information on stage at diagnosis since the late 1990s, preceding the substantial improvements in the availability of staging data in national cancer registration data in England from 2014 onwards. Data were collected initially by the Anglia Cancer Registry and subsequent organisations responsible for cancer registration (from April 2013 the National Cancer Registration and Analysis Service, which is part of Public Health England). For this study we used data from a historical cancer patient cohort, as in our previous research on stage at diagnosis of melanoma [12].

Stage at diagnosis was assigned by registry staff, based on clinical, imaging, and pathological information according to the TNM classification [13]. The 5th edition of TNM classification was used until 2010, and the 7th edition from 2011; differences between the two editions do not affect the classification of tumours in stage categories I-IV. Information was also available on year of diagnosis, sex, five-year age group, deprivation group, method of diagnosis (clinical/imaging, histology/cytology, unknown/other), last known survival status and the date this was recorded. We categorised deprivation according to quintiles of the income domain of the Index of Multiple Deprivation (IMD) [14]; we used IMD 2004 for diagnoses in 1999–2002; IMD 2007 for those in 2003–06; IMD 2010 for those in 2007–11; and IMD 2015 for those in 2012−16), using nationally defined cut-offs (1=least deprived; 5=most deprived).

### 2.2 Analysis

We calculated renal cancer incidence rates, treating the number of cases as the numerator and the estimated mid-year resident population of the former Anglia sub-region of the East of England as the denominator. Denominator population estimates were stratified by sex, age group, and IMD (see above) income deprivation quintile group. Year-specific denominator population estimates were used for each year except 2011−16, for which denominator population estimates from 2010 were used.

#### 2.2.1 Stage imputation

We imputed missing information on stage for 7.8 % (427/5,456) of all patients with multiple imputation prior to the main analyses, [15,16] using multinomial logistic regression. Independent variables were age-group, year of diagnosis, histological diagnosis status, and survival status and Nelson-Aalen estimator of the cumulative hazard for death at the latest observed datapoint (both censored at 1 year post-diagnosis). These were selected from a larger model which additionally included sex, IMD deprivation quintile, and Primary Care Trust;, none of which were significantly associated with missing stage. Ten imputed datasets were created, following the rule of thumb that their number should approximately correspond to percentage of missing data [17]. We carried out analyses on each of the ten datasets and used Rubin’s Rules to combine the results [18]. In sensitivity analysis, we carried out a ‘complete case analysis’ (CCA) including cases with non-missing stage only (n = 5,029).

Numbers and incidence rates: We first summarised counts of renal cancer cases by sex, age group, deprivation, and stage (including missing stage). We then examined age-standardised incidence rates of renal cancer by year of diagnosis, overall and by stage, using the imputed data. Rates were standardised to the European Standard Population 2013 [19]. Trends were plotted on the log-scale, to allow a fair representation of relative changes over time between stage groups with different baseline incidence. We plotted incidence trends separately by sex, consistent with other literature [16,20,21].

Incidence trends over time: Given no evidence of over-dispersion (Box S1), we used Poisson regression to assess overall and stage-specific temporal incidence trends, expressed as annual IRRs. These models were fitted overall and by stage category (i.e. five separate models), with year of diagnosis, sex, age group, and deprivation as main effects and a population off-set. For ‘diagnosis year’, segmentation was used to allow for appreciation of possible sub-period trends, if these provided superior model fit than estimating a single coefficient across the entire study period. Our aim was not to discover ‘true’ change-point(s), but to better estimate the shape of overall trends along the entire study period. An established method for doing so is using the US NCI/SEER Joinpoint software, [22,23] testing for best fit between non-segmented and different segmented models, given a maximum number of ‘change-points’, and for all possible combinations of change-points. Because Joinpoint does not cater for multiply imputed datasets we carried out this procedure manually, as detailed in Box S1. Consequently, the Stage I and the Stage 2 models were ‘segmented’ (at 2010 and 2003, respectively) as these change points provided optimal fit; the all-stages model, and the Stage II and Stage III models were not segmented as segmentation did not provide a better fit.

Incidence trends by patient group: We assessed whether overall and stage-specific incidence trends differed by patient group (sex, age and deprivation) by additionally including interaction terms sex*diagnosis year, age*diagnosis year and deprivation*diagnosis year, in turn, in the above described Poisson models. When inclusion of these pair-wise interaction terms was compared to main effects only using the log-likelihood ratio test, there was no evidence for interaction effects at the pre-specified 0.005 significance level. Therefore, higher order (e.g. three-way) interactions were not explored and IRRs by patient subgroups were not reported.

Given that we planned to test the significance of coefficients within several models, we considered a p-value less than 0.005 to represent statistical significance. All analyses were performed in either Stata v.13 (descriptive statistics, plots, and fitting final models) or R version 3.5.0 (finding optimal change-points – see Box S1).

## 3 Results

There were 5,456 cases of renal cancer diagnosed during 1999−2016 in our study population which ranged from 2,195,688 in the first study year (1999) to an estimated 2,472,283 in latter study years. Most patients were men (64 %) and aged 65 years or older (59 %) (Table S1). Among all patients, 32 %, 10 %, 19 %, and 31 % were diagnosed as Stages I, II, III, IV, respectively, while for 8% stage information was missing (see also Methods). During the study period the proportion of patients with observed Stage I increased (23% in 1999-2003 vs 39% in 2014-2016) while the proportion of patients in observed Stages IV or with missing stage decreased (37% in 1999-2003 vs 26% in 2014-2016).

**Fig. 1:**
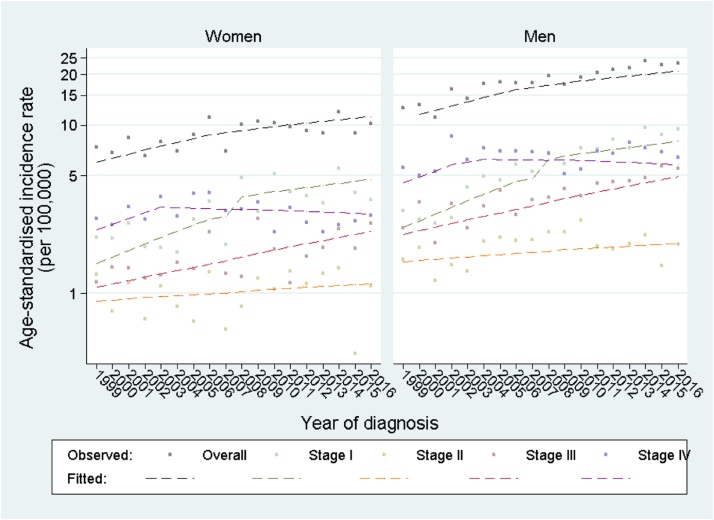
Age-standardised incidence of renal cancer (observed and fitted)*, overall and by stage at diagnosis, by sex. *Dashed lines estimated from five separate Poisson models (one for all cases, and one each for stage-specific cases) including main effect variables for sex, age group, deprivation, and year (segmented at 2010 for Stage I cases and 2003 for Stage IV cases). Exact model forms provided within Box S1. For 427 patients with missing values on stage, these values were imputed as described under ‘Multiple imputation’ in the Methods section.

The overall age-standardised incidence progressively increased during the study period, from 9.8 per 100,000 in 1999 to 16.4 per 100,000 in 2016 (Table S2). Incidence remained higher in men than women throughout the study period (both overall, and for stage-specific comparisons), with similar time-trends between sexes (Fig. 1). Increasing incidence trends for Stages I–III (and particularly Stage I) mirrored the overall increase in incidence trends (across all stage categories).

Poisson model estimates indicated that the annual IRR across all stages was 1.03 (95 % CI 1.03–1.04) (Table 1); that is, an approximate 3% increase in renal cancer incidence per year, concordantly with the observed patterns (Fig. 1). Stage I was associated with the fastest increasing trend, with annual IRR = 1.09 (95 % CI 1.07–1.12) during 1999−2010 and 1.03 (1.00–1.05) during 2011−2016. The increase in the incidence of Stage II and III was relatively slower (annual IRR = 1.01 (0.99, 1.03); and 1.03 (1.01, 1.06), respectively). In contrast, although Stage IV incidence increased during 1999−2003 with an annual IRR = 1.08 (1.02–1.13) it decreased continually thereafter (annual IRR = 0.99, 0.98–1.00 during 2004–2016).

**Table 1:** Adjusted annual Incidence Rate Ratios (95 % Confidence Intervals) of renal cancer*, overall and by stage of diagnosis.

	Time period 1		Time period 2**	
**Stage**	**1999−2016**			
**All**	1.03	(1.03, 1.04)		
	**1999−2010**		**2010−2016**	
**I**	1.09	(1.07, 1.12)	1.03	(1.00, 1.05)
	**1999−2016**			
**II**	1.01	(0.99, 1.03)		
	**1999−2016**			
**III**	1.03	(1.01, 1.06)		
	**1999−2003**		**2003−2016**	
**IV**	1.08	(1.02, 1.13)	0.99	(0.98, 1.00)

For 427 patients with missing values on stage (8% of entire dataset), these values were imputed as described under ‘Multiple imputation’ in the Methods section.

Coefficients for all main effects (including sex, age group, deprivation, and year) are presented for the model fitted to all cases, as Table S3. These coefficients were very similar for stage-specific models.

* Estimated from five separate Poisson models (one for all cases, and one each for stage-specific cases) including main effect variables for sex, age group, deprivation, and year (segmented at 2010 for Stage I cases and 2003 for Stage IV cases). Exact model forms provided within Box S1.

** Stage I IRRs and Stage IV IRRs indicated better fit when segmented at 2010 and 2003, respectively.

In sensitivity analysis including only cases with observed stage, time-trends were highly similar to those observed for multiply imputed data (Fig. S1).

## 4 Discussion

We observed consistently increasing incidence of renal cancer, driven by increasing incidence of Stage I disease, and in spite of declining incidence of Stage IV disease. There was no evidence of differences in incidence time-trends by sex, age, or deprivation group.

We used cancer registry data with highly complete information on stage at diagnosis, further complemented by use of multiple imputation to account for missing stage. We employed segmented regression techniques to improve the robustness of time trends estimates. The principal limitation of this study is that, although it provides information about incidence trends by stage, it does not illuminate the reasons underpinning them. Information on whether patients were diagnosed with symptoms of renal cancer or incidentally was not available; such information could elucidate whether increased incidental detection has contributed to increasing incidence of Stage I disease. We analysed data from the area of East Anglia, limiting the external validity of our findings. However, concerning the English population, no other region is served by highly complete staging information on incident cases of renal cancer for the study period of interest; therefore, our findings provide insights not otherwise available at a national level. The year 2010 was used for denominator information for 2011−2016. This should matter little for comparisons of stage-specific incidence trends against each other, but may have led to slight underestimation of absolute incidence for the study period.

Previous studies that described time-trends of renal cancer incidence typically do not consider stage-specific trends, cover substantially earlier eras, or do not employ robust statistical models. One study using US-SEER data for 1992–2015 that employed Joinpoint regression reported increasing incidence of localised disease until 2007 (plateauing thereafter) and decreasing incidence of distant renal cancer from 1998 onwards [8]. Although this previous study relates to a different era and country context, the reported time trends align with those observed in our study, though the observed percentage of cancer diagnosed at non-advanced stages was lower in our data. Our study substantially amplifies and updates previous UK evidence which covers earlier study eras and does not employ stage-specific analysis [1,20]; it considers more recent study periods, examining stage-specific time trends and potential changes in them. We observed a relative greater increase in the incidence of Stage III compared to that of Stage II, which may partly reflect secular trends in the assessment of pathology specimens.

Amongst stage-specific incidence rates of renal cancers in the past two decades, Stage I was associated with the biggest increases. This finding is compatible with two hypotheses. First, it may represent increasing rates of use of abdominal ultrasounds and CT scans during the study period, and thus rising number of incidental diagnoses [24,25]. Second, it may represent increasingly earlier detection of symptomatic disease, as indicated by decreasing incidence of Stage IV disease, which is least likely to be asymptomatic [26]. Prospective longitudinal research capturing the symptoms and investigation history of populations of patients diagnosed with renal cancer would be useful in quantifying the degree by which either hypothesis applies. System-wide efforts to improve earlier diagnosis of cancer over the last 25 years could indeed have contributed to the observed changes in stage-specific incidence trends [27]. The Be Clear on Cancer ‘blood in pee’ campaigns (delivered nationally from 2013 onwards), are unlikely to have contributed to the trends observed here, as they would only cover the last few years of our study period. However, they may contribute to further reductions in rates of late-stage disease in future [28]. Our findings support examining stage-specific results as part of future evaluations of these campaigns and other early diagnosis interventions.

The notable increase in the incidence of renal cancer overall was mirrored by that for early-stage disease. This finding is compatible with potential increasing incidental identification of some renal cancers. However, the accompanying decrease in incidence of late-stage disease suggests progressively earlier diagnosis of symptomatic cases.

## Authorship contribution statement

AH, MEB, MMK, GAA were responsible for statistical analysis. BR provided commentary on stage registration. The study was originally conceived by DCG and GL. All authors commented on study design, interpretation and commented on the manuscript

## Declaration of Competing Interest

AH, MEB, MMK, BR, DCG, GAA, and GL have no conflicts of interest to declare.
