# Supplementary material for: Stage–specific incidence trends of renal cancers in the East of England, 1999–2016
Source: Cancer Epidemiol. 2021 Apr;71(Pt A):101883. doi: 10.1016/j.canep.2020.101883 (PMC7988458; doi:10.1016/j.canep.2020.101883)
Supplement: Supplementary file 1 [file mmc1.docx]

Supporting Information

**Box S1: Model selection process**

We fitted a segmented Poisson model (also known as a ‘piecewise’ model) for all cases and by each stage, given that the plotted stage-specific incidence trends (Figure 1) were suggestive of the presence of potential ‘change-points’ (or ‘break-points’).^[[1]](#footnote-1)^^[[2]](#footnote-2)^ The approach was similar to that of SEER’s Joinpoint software,^[[3]](#footnote-3)^ testing for the best fit between a non-segmented model and different segmented models (given a maximum number of ‘change-points’, and for all possible combinations of change-points), via permutation. Given that Joinpoint is not currently available for dealing with multiply imputed data, we carried out a similar process in the following steps*:

1. **For models fitted to all cases overall, and per stage, we found the optimal change-points for a model including a single change-point ([1]), and a model including a pair of change-points ([2]).**

$\log\left( \frac{n_{y}}{p_{y}} \right)= \beta_{0}+\beta_{1i}{agegroup}_{i}+\beta_{2}male+\beta_{3_{j}}{deprivation}_{j}+\beta_{4}t_{1999}+\beta_{5}t_{c}+\varepsilon$ [1]

where $\beta$’s are coefficients, $n_{y}$ is the number of renal cancer cases in year y, $p_{y}$ is the denominator population in year y, $t_{1999}$ is the number of time-points since 1999, $t_{c}$ is number of time-points since change-point c, and $\varepsilon$ is the error (residual) term $\left( y=1999,\ldots2016 [note y = 1999+ t_{1999}+ t_{c}]; i=1,\ldots8; j=1,\ldots4; c=2000,\ldots2015 \right)$

$\log\left( \frac{n_{y}}{p_{y}} \right)= \beta_{0}+\beta_{1i}{agegroup}_{i}+\beta_{2}male+\beta_{3_{j}}{deprivation}_{j}+\beta_{4}t_{1999}+\beta_{5}t_{c_{1}}+\beta_{6}t_{c_{2}}+\varepsilon$ [2]

where $\beta$’s are coefficients, $n_{y}$ is the number of renal cancer cases in year y, $p_{y}$ is the denominator population in year y, $t_{1999}$ is the number of time-points since 1999, $t_{c_{1}}$is number of time-points since change-point c_1_, $t_{c_{2}}$ is number of time-points since change-point c_2_, and $\varepsilon$ is the error (residual) term $\left( y=1999,\ldots2016 [note y = 1999 + t_{1999}+t_{c_{1}}+t_{c_{2}}]; i=1,\ldots8; j=1,\ldots4; c_{1}=2000,\ldots2014; c_{2}=2001,\ldots2015 \right)$

To determine the optimal single change-points (one per stage), we compared the median Akaike’s Information Criteria (AIC) value between the 16 possible change points (each year from 2000 to 2015). We firstly, per change-point, calculated the median AIC across the (same) individual models fitted within each of the ten imputed datasets. We then compared the 16 resulting median AICs. The median AIC was lowest (indicating best model fit) when the change-points were 2006 (all-stage model), 2010 (Stage-I), 2010 (Stage-II), 2009 (Stage-III), and 2003 (Stage-IV). We used a similar method to determine the possible presence of two optimal change-points (i.e. 120 possible pairs for a maximum 16 change points for all years other than the first/last study years) per stage: optimal pairs of change-points were 2000 and 2003 (all-stage model), 2006 and 2008 (Stage I), 2000 and 2010 (Stage II), 2010 and 2011 (Stage III), and 2000 and 2002 (stage-IV).

We studied a maximum of two change-points as the plotted data suggested one to two potentially different change-points between stages (e.g. an initial acceleration in Stage I incidence, followed by a deceleration in later study years; a gradual increase in Stage III incidence with little change in slope over time).

1. **Overall and within stage, compared the fit of a model with no change-point ([3]) to a model with the optimal single change-point ([1]), and a model with the optimal single change-point ([1]) to a model with the optimal pair of change-points ([2]).**

$\log\left( \frac{n_{y}}{p_{y}} \right)= \beta_{0}+\beta_{1i}{agegroup}_{i}+\beta_{2}male+\beta_{3_{j}}{deprivation}_{j}+\beta_{4}y +\varepsilon$ [3]

where $\beta$’s are coefficients, $n_{y}$ is the number of renal cancer cases in year y, $p_{y}$ is the denominator population in year y, y is the year of diagnosis, and $\varepsilon$ is the error (residual) term $(y=1999,\ldots2016; i=1,\ldots8; j=1,\ldots4)$

We compared the three models (no, single, and pair of change-points) where the coefficient estimates had been derived via Rubin’s rules. Log-likelihood tests indicated that a single change-point model was a significantly better fit to the data than a model with no change point in the case of modelling Stage I cases (p=0.001) and Stage IV cases (p=0.004), but not overall or for Stage II or III cases (p ranged from 0.03 to 0.45). No log-likelihood tests indicated that a model including two change-points was a significantly better fit than a model with a single change-point (p ranged from 0.04 to 1.00). One of the p-values was 0.04 (for Stage I cases when comparing a model of two change-points vs. a model of one change-point; i.e. significant at the ‘traditional’ 0.05 level), this was not below our pre-specified significance level of 0.005, which had been set as such to account for the multiple testing during both the model selection process and later when interpreting final model coefficients.

1. **Re-fit the best-fitting models from 2. also including an interaction sex*year**

When an interaction term sex*year was included in the best-fitting models so far (all Poisson models, overall: no change-point; Stage I: single change-point at 2010; Stage II: no change-point; Stage 3: no change-point; Stage IV: single change-point at 2003) this term was not statistically significant at the 0.005 level for any of these models (p ranged from 0.008 to 0.7).

**The final models fitted were:**

Overall: a segmented Poisson model with no change-point

Stage I: a segmented Poisson model with one change-point (2010)

Stage II: a Poisson model with no change-point

Stage III: a Poisson model with no change-point

Stage IV: a segmented Poisson model with one change-point (2003)

*We did not re-fit Negative Binomial models as is sometimes done using Joinpoint due to a lack of over-dispersion of the data.

**Table S1: Demographic and clinical characteristics of primary renal cancer cases diagnosed in East of England during 1999–2016 (split into five– year periods except for 2014-16)**

|  | **1999–2003** | | **2004–08** | | **2009–13** | | | **2014-2016** | | | **1999–2016**  **(total study period)** | |  |
| --- | --- | --- | --- | --- | --- | --- | --- | --- | --- | --- | --- | --- | --- |
| *Total* | 1053 | (100.0) | 1470 | (100.0) | | 1747 | (100.0) | | 1186 | (100.0) | 5,456 | (100.0) | |
| *Sex* |  |  |  |  | |  |  | |  |  |  |  | |
| Female | 412 | (39.1) | 528 | (35.9) | | 620 | (35.5) | | 393 | (33.1) | 1,953 | (35.8) | |
| Male | 641 | (60.9) | 942 | (64.1) | | 1127 | (64.5) | | 793 | (66.9) | 3,503 | (64.2) | |
| *Age group (years)* |  |  |  |  | |  |  | |  |  |  |  | |
| 25–49 | 125 | (13.5) | 173 | (13.3) | | 213 | (13.9) | | 171 | (16.8) | 682 | (14.3) | |
| 50–54 | 93 | (10.0) | 93 | (7.2) | | 125 | (8.1) | | 86 | (8.5) | 397 | (8.3) | |
| 55–59 | 97 | (10.5) | 159 | (12.3) | | 139 | (9.1) | | 118 | (11.6) | 513 | (10.7) | |
| 60–64 | 122 | (13.1) | 176 | (13.6) | | 235 | (15.3) | | 139 | (13.7) | 672 | (14.1) | |
| 65–69 | 152 | (16.4) | 180 | (13.9) | | 275 | (17.9) | | 175 | (17.2) | 782 | (16.4) | |
| 70–74 | 159 | (17.1) | 190 | (14.6) | | 227 | (14.8) | | 159 | (15.7) | 735 | (15.4) | |
| 75–79 | 146 | (15.7) | 257 | (19.8) | | 229 | (14.9) | | 145 | (14.3) | 777 | (16.3) | |
| 80–84 | 103 | (11.1) | 154 | (11.9) | | 178 | (11.6) | | 117 | (11.5) | 552 | (11.6) | |
| 85+ | 56 | (6.0) | 88 | (6.8) | | 126 | (8.2) | | 76 | (7.5) | 346 | (7.2) | |
| *Deprivation (IMD quintile)* |  |  |  |  | |  |  | |  |  |  |  | |
| Least | 209 | (19.8) | 259 | (17.6) | | 316 | (18.1) | | 250 | (21.1) | 1034 | (19.0) | |
| 2 | 272 | (25.8) | 421 | (28.6) | | 501 | (28.7) | | 334 | (28.2) | 1528 | (28.0) | |
| 3 (Middle) | 301 | (28.6) | 424 | (28.8) | | 510 | (29.2) | | 312 | (26.3) | 1,547 | (28.4) | |
| 4 | 185 | (17.6) | 249 | (16.9) | | 291 | (16.7) | | 183 | (15.4) | 908 | (16.6) | |
| Most | 86 | (8.2) | 117 | (8.0) | | 129 | (7.4) | | 107 | (9.0) | 439 | (8.0) | |
| *Stage at diagnosis* |  |  |  |  | |  |  | |  |  |  |  | |
| I | 238 | (22.6) | 445 | (30.3) | | 605 | (34.6) | | 466 | (39.3) | 1,754 | (32.1) | |
| II | 113 | (10.7) | 157 | (10.7) | | 177 | (10.1) | | 99 | (8.3) | 546 | (10.0) | |
| III | 180 | (17.1) | 256 | (17.4) | | 333 | (19.1) | | 259 | (21.8) | 1028 | (18.8) | |
| IV | 384 | (36.5) | 509 | (34.6) | | 501 | (28.7) | | 307 | (25.9) | 1,701 | (31.2) | |
| Missing | 138 | (13.1) | 103 | (7.0) | | 131 | (7.5) | | 55 | (4.6) | 427 | (7.8) | |

Data presented as n (column percentage)

**Table S2: Age-standardised incidence rates in East of England in 1999 and 2016, overall and stage-specific**

| **Population** | **Stage** | **Age-standardised incidence rate (cases per 100,000)** | | |
| --- | --- | --- | --- | --- |
|  |  | **1999** | **2007** | **2016** |
| All | All | 9.8 | 12.0 | 16.4 |
|  | I | 2.5 | 3.5 | 6.3 |
|  | II | 1.5 | 1.3 | 1.5 |
|  | III | 1.8 | 2.4 | 4.0 |
|  | IV | 4.1 | 4.8 | 4.6 |
| Women | All | 7.4 | 7.0 | 10.2 |
|  | I | 2.1 | 2.0 | 3.6 |
|  | II | 1.3 | 0.6 | 1.1 |
|  | III | 1.2 | 1.3 | 2.6 |
|  | IV | 2.8 | 3.1 | 2.9 |
| Men | All | 12.7 | 17.9 | 23.4 |
|  | I | 3.1 | 5.3 | 9.5 |
|  | II | 1.6 | 2.1 | 2.0 |
|  | III | 2.4 | 3.6 | 5.5 |
|  | IV | 5.6 | 6.9 | 6.4 |

For 427 patients with missing values on stage (7.8% of the entire sample), these values were imputed as described under ‘Multiple imputation’.


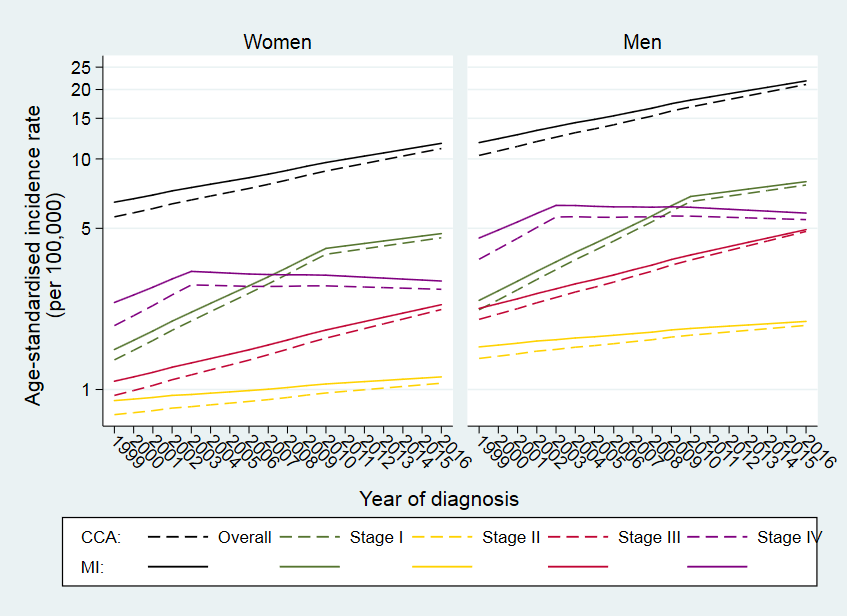


**Figure S1: Predicted age-standardised incidence of renal cancer (including when stage imputed and not), by stage at diagnosis and sex**

Lines estimated from a Poisson model including main effect variables for sex, age group, deprivation, and year (segmented at 2007 overall, 2006 and 2008 for stage I, 2000 and 2010 for stage II, and 2000 and 2002 for stage IV). In the case of stage II, the model also included an interaction term sex*year.

MI = Multiple imputation (for 427 patients with missing values on stage, these values were imputed as described under ‘Multiple imputation’ in the Methods section). CCA = Complete Case Analysis (analyses carried out on patients with non-missing values on stage).

**Table S3: Coefficient estimates for model fitted to all renal cancer cases***

|  | **IRR** | **(95% CI)** | **p-value**† |
| --- | --- | --- | --- |
| Sex (ref female) |  |  |  |
| Male | 2.05 | (1.94, 2.16) | <0.0001 |
| Age group (ref 65-69y) |  |  |  |
| 25-49 | 0.09 | (0.08, 0.10) | <0.0001 |
| 50–54 | 0.39 | (0.35, 0.44) | <0.0001 |
| 55–59 | 0.53 | (0.47, 0.59) | <0.0001 |
| 60–64 | 0.70 | (0.63, 0.78) | <0.0001 |
| 65–69 | (ref) |  |  |
| 70–74 | 1.11 | (1.00, 1.23) | 0.0459 |
| 75–79 | 1.45 | (1.31, 1.60) | <0.0001 |
| 80–84 | 1.44 | (1.29, 1.60) | <0.0001 |
| 85+ | 1.07 | (0.94, 1.21) | 0.2960 |
| *Deprivation (ref Least deprived)* |  |  |  |
| Quintile 2 | 1.10 | (1.01, 1.19) | 0.0215 |
| Quintile 3 | 1.05 | (0.97, 1.13) | 0.2585 |
| Quintile 4 | 1.07 | (0.98, 1.17) | 0.1354 |
| Most deprived | 1.01 | (0.91, 1.13) | 0.8304 |
| Year of diagnosis | 1.03 | (1.03, 1.04) | <0.0001 |

CI = Confidence Interval; dp =decimal places, IRR = Incidence Rate Ratio; ref = Reference category

*Model fitted is described in Box S1.

For 427 patients with missing values on stage (8% of the entire sample), these values were imputed as described under ‘Multiple imputation’ in the Methods section.

†Null hypothesis: $\beta$ = 0

1. Muggeo VM. Estimating regression models with unknown break-points. Stat Med. 2003;22(19):3055-71. [↑](#footnote-ref-1)
2. Kim HJ, Fay MP, Feuer EJ, Midthune DN. Permutation tests for joinpoint regression with applications to cancer rates. Stat Med. 2000;19(3):335-51. [↑](#footnote-ref-2)
3. National Cancer Institute. Division of Cancer Control & Population Sciences. Joinpoint Trend Analysis Software. 2019 [Available from: <https://surveillance.cancer.gov/joinpoint/>. [↑](#footnote-ref-3)
